# No Effect of Added Sugar Consumed at Median American Intake Level on Glucose Tolerance or Insulin Resistance

**DOI:** 10.3390/nu7105430

**Published:** 2015-10-23

**Authors:** Joshua Lowndes, Stephanie S. Sinnett, James M. Rippe

**Affiliations:** 1 Rippe Lifestyle Institute, 215 Celebration Place, Suite 300, Celebration, FL 34747, USA; jlowndes@rippelifestyle.com (J.L.); rdsinnett@gmail.com (S.S.S.); 2 Rippe Lifestyle Institute, 21 N. Quinsigamond Avenue, Shrewsbury, MA 01545, USA; 3 Department of Biomedical Sciences, University of Central Florida, Orlando, FL 32826, USA

**Keywords:** fructose, sucrose, glucose, high fructose corn syrup, diabetes

## Abstract

Excess sugar consumption may promote adverse changes in hepatic and total body insulin resistance. Debate continues over the effects of sugars at more typically consumed levels and whether the identity of the sugar consumed is important. In the present study participants (20–60 years old) were randomly assigned to one of five groups, three that consumed low fat milk with added fructose containing sugars in amounts equivalent to the 50th percentile of fructose consumption (US), one which consumed low-fat milk sweetened with glucose, and one unsweetened low-fat milk control group. The intervention lasted ten weeks. In the entire study population there was less than 1 kg increase in weight (73.6 ± 13.0 *vs.* 74.5 ± 13.3 kg, *p* < 0.001), but the change in weight was comparable among groups (*p* > 0.05). There were no changes in fasting glucose (49 ± 0.4 *vs.* 5.0 ± 0.5 mmol/L), insulin (56.9 ± 38.9 *vs.* 61.8 ± 50.0 pmol/L), or insulin resistance, as measured by the Homeostasis Model Assessment method (1.8 ± 1.3 *vs.* 2.0 ± 1.5, all *p* > 0.05). These data suggest that added sugar consumed at the median American intake level does not produce changes in measures of insulin sensitivity or glucose tolerance and that no sugar has more deleterious effects than others.

## 1. Introduction

Diabetes has grown in parallel with the worldwide [1,2,3,4] increase in obesity and is widely linked to it. Diabetes is also strongly linked to other diseases which have a metabolic component, such as coronary heart disease (CHD) and metabolic syndrome (MetS). This has prompted scientists to explore a number of dietary factors in the development and progression of cardiometabolic disease. Some of the nutritional factors that have been implicated have been sugars, particularly those containing fructose, including fructose itself, high fructose corn syrup (HFCS), and sucrose [5,6,7,8,9,10,11,12,13,14,15]. Evidence supporting a putative link between fructose containing sugars and diabetes comes from ecologic studies [9,10] which compare the availability of sugars to the prevalence of diabetes, animal studies [16,17,18,19,20,21], and studies employing either a fructose *vs.* glucose model or utilizing doses of fructose-containing sugars which are often four to five times the level found in the human diet [22].

Higher quality evidence from randomized controlled trials, systematic reviews, meta-analyses, and prospective cohort studies, generally have not supported a link between fructose-containing sugars and the development of type 2 diabetes [23,24,25,26,27,28,29]. To our knowledge, no study has specifically compared fructose-containing sugars that are normally consumed in the human diet (*i.e.*, HFCS and sucrose) *vs.* fructose by itself with glucose and no added sugar controls. This is important for several reasons. Firstly, the combination of fructose and glucose consumed together may alter the metabolism of fructose compared to fructose alone. Secondly, it has been argued that it is the fructose moiety in HFCS and sucrose that may lead to a variety of metabolic abnormalities which suggests that this may be different than glucose. The proposed mechanism for linkage between fructose and diabetes is based on the postulated linkage between fructose and insulin resistance [12]. One proposed mechanism for fructose-induced insulin resistance is that fructose consumption may increase *de novo* lipogenesis (DNL) leading to increased fat in the liver and hepatic insulin resistance [30,31]. Data to support these assertions, however, is limited and, as already indicated, comes from studies that were given very large doses of fructose or glucose, which are typically not consumed in isolation in the human diet. Since the issues of insulin resistance are important, and much of the previous data comes largely from artificial experiments where either fructose or glucose were administered alone and often in very large doses (e.g., 25% of calories) [22], as a practical matter we felt it was important to determine whether or not normally-consumed levels of added sugar consumption created similar abnormalities. Moreover, the degree to which the amount of DNL in humans consuming sugars or other carbohydrates typically found in the human diet suggests that it is a very limited pathway comprising no more than 1%–5% of consumed fructose, which then may be turned into triglycerides [32,33].

Accumulating evidence has established that insulin resistance plays a major role in the development of diabetes [34,35]. Typically, insulin resistance precedes the onset of diabetes by 10–20 years. Moreover, prospective studies have demonstrated that insulin resistance is the best predictor of whether or not an individual will develop diabetes [34,35]. Thus, if a significant linkage exists between fructose containing sugars at normal typical levels of fructose consumption and in the sugars typically found in the human diet (e.g., sucrose or HFCS) and insulin resistance, this would carry significant implications for nutritional recommendations.

The purpose of the current study was to explore whether fructose containing sugars (fructose itself, HFCS, and sucrose) consumed at the median level of American intake increased insulin resistance or decreased glucose tolerance when compared to a glucose control and an unsweetened beverage control. Our research group had previously demonstrated that various levels of consumption of fructose and glucose containing sugars (sucrose and HFCS) ranging from the 25th to the 90th percentile population consumption of fructose did not increase risk of diabetes [36]. The current study expands these findings by including pure fructose and glucose conditions as well as an unsweetened control.

Our hypothesis was that median American intake levels of fructose containing sugars (fructose itself, HFCS, and sucrose) would have no effect on either glucose tolerance or insulin resistance and would not differ from a glucose control or unsweetened beverage control.

## 2. Methods

### 2.1. Overview

This was a randomized, prospective, parallel group, partially-blinded study to assess the effects of incorporation of various sugars into the diet by drinking sugar sweetened low-fat milk or a control, unsweetened milk (Table 1). Subjects were blinded concerning which level and type of sugar they were consuming. Staff members also were blinded as to which sugar subjects were consuming, but needed to be aware of whether subjects were consuming 18% or 9% of calories from added sugar in order to prescribe the rest of the diet. Since our goal was to look at the median consumption of fructose, which is in the American population 9% of calories, it was necessary to prescribe 18% of calories from HFCS-55 or sucrose since that would provide us essentially 9% of calories from fructose given that these two sugars are composed of roughly half fructose and half glucose. The level of 9% of calories from glucose was chosen to equilibrate the energy to the 9% of calories from fructose. One of our goals of the study was to compare fructose containing sugars (e.g., HFCS, sucrose and fructose) to a glucose control. To put this in perspective, for an individual consuming a 2000 kcal diet this would mean that 45 grams of fructose or glucose were included in the daily diet or 90 grams of either HFCS or sucrose. The control condition of unsweetened milk was chosen to serve as a control to all of the sugar conditions.

The study had a duration of ten weeks. The study was approved by the Western Institutional Review Board (WIRB protocol number 20131024; approval date: 20 June 2013). This study has been registered on the National Institute of Health (NIH) Clinical Trials website: NCT02278042.

**Table 1:** Study design *.

Group	Nutritional Interventions
Group 1	HFCS sweetened milk with HFCS contributing 18% of estimated weight-maintenance energy intake
Group 2	Fructose sweetened milk with fructose contributing 9% of estimated weight-maintenance energy intake
Group 3	Glucose sweetened milk with glucose contributing 9% of estimated weight-maintenance energy intake
Group 4	Sucrose sweetened milk with sucrose contributing 18% of estimated weight-maintenance energy intake
Group 5	Total calories in unsweetened milk contributes 9% of estimated weight-maintenance energy intake

* Milk consumption was determined by the amount of calories required for weight-maintenance; HFCS, high fructose corn syrup.

### 2.2. Study Population

The study population included men and women between the ages of 20 and 60 years, with a body mass index (BMI) between 21 and 35 kg/m^2^. Thus, the study cohort contained individuals who were “healthy” weight, overweight, and obese. Participants were recruited from a database of previous research participants in addition to advertisements in local newspapers and social media. All participants were weight-stable (no change in weight greater than 3% in the past month, no actions taken in three months to lose weight), non-smokers (not been a regular smoker for at least twelve months and no social smoking for at least three months) and normoglycemic (based on fasting and after a two-hour oral glucose challenge in a smaller subpopulation). Participants were excluded if they had uncontrolled blood pressure, a history of thyroid disease, cancer, gastrointestinal disorders, cardiac problems, eating disorders, if they had ever had a surgical procedure for weight loss, if they had undergone any major surgical procedure in the previous three months, if they started a new medication within the past three months (including a change in dose of an existing medication), if they were pregnant or lactating, if they consumed more than three alcoholic drinks per week or if they had any significant food allergy.

Additionally, participants were not allowed to enroll if they had participated in any other clinical trial within the previous 30 days. All participants provided signed informed consent.

### 2.3. Intervention

The intervention required 10 weeks of daily consumption of low-fat milk as part of a usual diet. Milk was sweetened with one of four types of sugar—fructose, glucose, sucrose or HFCS, and a fifth group that contained unsweetened low-fat milk to act as a control for milk consumption (see Table 1). The number of servings per day was linked to the estimated amount of calories required for weight maintenance. The estimation was made using the Mifflin, St. Jeor Equation [37] and using an appropriate activity factor determined from responses to a physical activity questionnaire. Glucose and fructose sweetened milk was consumed in amounts that the added sugar contributed 9% of the estimated weight maintenance calories. Sucrose and HFCS sweetened milk was consumed in amounts that the added sugar contributed 18% of the estimated weight maintenance calories. The unsweetened control milk was consumed in amounts that the total calories from the milk contributed 9% of the estimated weight maintenance calories.

Participants were provided information on how to account for the calories in the milk, but otherwise told to eat their usual diet. Diets for each individual were assessed at the beginning and at the end of the protocol by research nutritionists trained in the University of Minnesota Nutrition Data System for Research. This system was utilized to assess both usual diet and changes in diet.

Feedback on body weight was provided at weekly weigh-ins, but no instruction was given in the event of weight gain. The weekly in-clinic weigh-ins were also used to check compliance with a review of milk consumption checklists and to provide another week’s supply of milk.

Participants were randomly assigned to their group according to the first unallocated number in a random sequence generated by an online random sequence generator. Group allocation occurred at completion of the pre-testing phase and was conducted by the clinical manager. The randomization sequence was generated by the study coordinator and was not accessible by the recruiter. No restrictions were placed on random group assignment. In addition, the clinical manager was blinded to the identity of the sugars in the respective groups.

### 2.4. Oral Glucose Tolerance Test and Blood Plasma Measurements

A standard two-hour Oral Glucose Tolerance Test (OGTT) was performed during pre-testing and again after completion of the ten week intervention on a sub-group of subjects who were also undergoing additional measurements in our metabolic unit. Prior to consumption of the 75 g glucose solution, an intravenous line was inserted and a blood sample was obtained for the measurement of fasting glucose and insulin. Participants were then given the glucose solution and allowed five min to consume it. Additional blood samples were then obtained after 30, 60, 90, and 120 min. At all-time points blood was collected in BD vacutainers containing ethylenediaminetetraacetic acid (EDTA) for the preparation of plasma. Aliquots from collected samples were obtained and divided into two. The first aliquot was used immediately to measure glucose using the YSI 2300 analyzer (YSI Incorporated, Yellow Springs, OH, USA). The remaining aliquot was stored at −80 °C for future batch testing of insulin via ELISA with kits EZHI-14K from EMD Millipore (Darmstadt, Germany).

### 2.5. Derived Measurements

Various measures of glucose homeostasis were derived from the fasting values of glucose and insulin, and from values at various time points during the OGTT. Homeostasis Model Assessment of Insulin Resistance was calculated from the fasting insulin and glucose measurements—(Glucose XInsulin)/22.5) [38]. Two hour Area Under the Curve (AUC) values were calculated for glucose and insulin using the standard trapezoidal method. Hepatic insulin resistance was measured as the product of glucose and insulin values at 30 min of the OGTT, a procedure validated against clamp methods [39]. Whole body insulin sensitivity and hepatic insulin resistance were calculated from samples obtained during the OGTT using the Matsuda Insulin Sensitivity Index (ISI) [40].

### 2.6. Statistical Analyses

All data are presented as means ± standard deviation (SD) and analyzed using SPSS-PASW Statistics version 18.0 (IBM, Armonk, NY, USA). Outcome measures were analyzed via a 5 (group type) × 2 (time) analysis of variance (ANOVA) with repeated measures. Significant time by group interactions resulted in two additional analyses. Firstly, the effects of all sugar sweetened milk combined was tested against the control milk using a 2 × 2 ANOVA with repeated measures. Secondly, all groups were treated individually—paired sample *t*-tests were performed for the pre and post values in each group separately and the difference between pre and post values were calculated and analyzed in a one way ANOVA with Tukey’s *post hoc* for pairwise comparisons, as necessary. Statistical significance was defined by *p* < 0.05.

## 3. Results

### 3.1. Demographic Information

**Figure 1:**
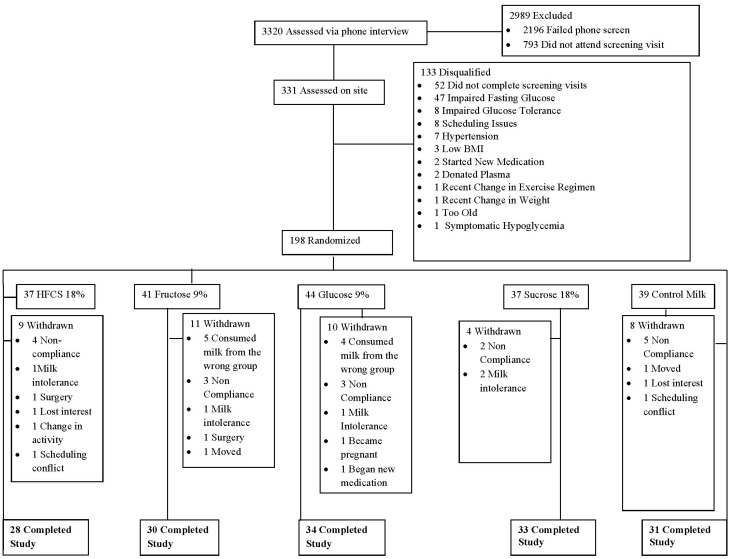
Participant flow through the various phases of the study.

A total of 331 prospective participants were screened on site, of which 198 qualified and completed all the pre-testing procedures (Figure 1). Table 2 presents subject characteristics of the 156 participants who completed the study and on whom further data are presented. There was a baseline difference in total cholesterol among the groups, but *post hoc* analysis failed to reveal any significant pairwise differences. Post-test fasting blood samples were only available on 152 participants and OGTTs were only completed on a sub-group of 93 participants, as already indicated.

### 3.2. Dietary Intake

Energy intake increased in all five groups by between 200–400 kcals/day. While all of these measurements changed from pre to post (pre < 0.001), there were no differences among the five groups (interaction 0.136). The increase in energy intake was driven largely by increase in carbohydrate, in general, and, in particular, total sugar and added sugar. As expected, both total sugar and added sugar in the HFCS 18% and sucrose 18% were different from the fructose 9%, glucose 9% and control conditions. These data are shown on Table 3.

**Table 2:** Baseline characteristics of the 156 participants who completed the intervention.

	HFCS 18% *n* = 28	Fructose 9% *n* = 30	Glucose 9% *n* = 34	Sucrose 18% *n* = 33	Control *n* = 31	All	*p*
Age (years)	36.5 ± 11.3	35.6 ± 10.4	37.0 ± 11.7	34.1 ± 11.0	35.3 ± 12.5	35.7 ± 11.4	0.864
Gender	*M* = 11, *F* = 17	*M* = 16, *F* = 14	*M* = 17, *F* = 17	*M* = 15, *F* = 18	*M* = 10, *F* = 21	*M* = 69, *F* = 87	
Weight (kg)	73.3 ± 13.2	74.3 ± 13.1	76.2 = 12.2	72.1 ±14.1	72.3 ± 12.2	73.7 ± 12.9	0.696
BMI	26.5 ± 3.5	26.0 ± 3.8	26.5 ± 3.3	25.6 ± 3.5	25.6 ± 3.6	26.0 ± 3.5	0.740
Waist Circumference (cm)	81.8 ± 10.7	81.3 ± 10.6	82.4 ± 10.1	80.8 ± 10.1	79.1 ± 8.8	81.1 ± 10.0	0.757
Systolic Blood Pressure (mmHg)	107.3 ± 11.1	105.8 ± 10.6	105.8 ± 8.7	107.2 ± 9.5	107.3 ± 9.0	106.7 ± 9.7	0.931
Diastolic Blood Pressure (mmHg)	68.5 ± 8.5	68.7 ± 7.2	68.1 ± 8.2	68.8 ± 7.7	69.6 ± 8.1	68.7 ± 7.9	0.957
Cholesterol (mmol/L)	4.9 ± 0.7	4.4 ± 0.8	5.0 ± 1.0	4.5 ± 0.9	4.4 ± 0.9	4.6 ± 0.9	0.022
Triglycerides (mmol/L)	1.3 ± 0.8	1.1 ± 0.7	1.4 ± 1.4	1.1 ± 0.6	0.8 ± 0.4	1.1 ± 0.9	0.121
HDL (mmol/L)	1.4 ± 0.4	1.3 ± 0.4	1.4 ± 0.4	1.4 ± 0.3	1.4 ± 0.5	1.4 ± 0.4	0.452
LDL (mmol/L)	2.9 ± 0.7	2.6 ± 0.6	2.9 ± 0.9	2.6 ± 0.8	2.6 ± 0.7	2.7 ± 0.8	0.160
Glucose (mmol/L)	5.1 ± 0.4	4.9 ± 0.4	5.0 ± 0.4	4.8 ± 0.4	5.0 ± 0.4	4.9 ± 0.4	0.058
Insulin (pmol/L)	67.4 ± 38.9	55.6 ± 31.9	55.6 ± 52.1	54.9 ± 33.3	54.2 ± 35.4	57.6 ± 38.9	0.687

Note: Baseline difference revealed among the groups, but *post hoc* analysis (Tukey’s) failed to reveal any significant pairwise differences between any two groups; HFCS, high fructose corn syrup; M, male; F, female; BMI, body mass index; HDL, high-density lipoprotein; LDL, low-density lipoprotein.

**Table 3:** Dietary intake.

		All	Time	HFCS 18%	Fructose 9%	Glucose 9%	Sucrose 18%	Control Milk	Interaction
Energy Intake (kcal)	Pre	1994.5 ± 692.6	<0.001	1897.4 ± 689.2	1988.1 ± 659.9	2031.7 ± 644.1	2050.6 ± 819.5	1979.9 ± 669.1	0.136
	Post	2296.0 ± 669.0		2384.0 ± 598.4	2198.6 ± 691.7	2189.0 ± 524.1	2492.0 ± 835.3	2229.1 ± 637.3	
Fat (g)	Pre	74.5 ± 30.5	0.837	70.4 ± 28.1	74.3 ± 23.4	75.2 ± 31.6	78.1 ± 39.2	73.4 ± 28.1	0.482
	Post	73.8 ± 27.1		69.4 ± 21.2	71.8 ± 26.3	67.7 ± 21.4	80.8 ± 37.3	78.9 ± 24.2	
Carbohydrate (g)	Pre	247.3 ± 92.6	<0.001	232.0 ± 99.3	250.7 ± 100.8	256.3 ± 79.8	249.1 ± 93.6	244.6 ± 95.9	0.002
	Post	305.2 ± 92.7		337.4 ± 93.1 ***	286.9 ± 81.7 ^¥^	297.3 ± 74.8 *^,¥^	333.4 ± 106.2 ***	275.0 ± 95.0 ^¥^	
Protein (g)	Pre	88.7 ± 41.1	<0.001	83.6 ± 35.3	86.0 ± 41.3	88.4 ± 33.5	93.9 ± 50.4	90.4 ± 44.3	0.834
	Post	109.2 ± 43.0		108.4 ± 36.8	108.7 ± 59.9	103.8 ± 30.9	114.4 ± 47.8	110.9 ± 36.2	
Total Sugar (g)	Pre	103.0 ± 51.3	<0.001	96.0 ± 48.7	102.8 ± 57.8	107.7 ± 44.2	100.6 ± 45.7	106.3 ± 61.7	<0.001
	Post	174.4 ± 58.1		207.0 ± 58.9 ***	155.7 ± 38.6 ***^,†^	170.2 ± 42.8 ***^,†^	204.5 ± 56.4 ***	137.9 ± 62.1 **^,†^	
Added Sugar (g)	Pre	56.5 ± 38.5	<0.001	49.3 ± 38.1	56.7 ± 35.5	59.5 ± 38.4	55.1 ± 35.6	60.3 ± 45.6	<0.001
	Post	98.2 ± 46.5		131.2 ± 32.8 ***	82.3 ± 23.8 **^,†^	93.8 ± 33.7 ***^,†^	132.0 ± 44.4 ***	54.8 ± 44.4 ^†,Δ^	

Note: ** different than baseline, *p* < 0.01; *** different than baseline, *p* < 0.005; ^¥^ change different than 18% HFCS; ^†^ change from baseline different than both 18% groups; ^Δ^ change from baseline different than both 9% groups.

### 3.3. Body Weight and Fasting Measures

In the entire pooled study population body weight changed less than 1 kg, but this was statistically significant (73.6 ± 13.0 *vs.* 74.5 ± 13.3 kg, *p* < 0.001). However, there were no differences among the five groups with respect to change in weight. Fasting glucose (4.9 ± 0.4 *vs.* 5.5 ± 0.5 mmol/L) and insulin (56.9 ± 38.9 *vs.* 61.8 ± 50.0 pmol/L) values were both unchanged, as was the HOMA measure of insulin resistance (1.8 ± 1.3 *vs.* 2.0 ± 1.5, all *p* > 0.05). In all cases the time by group interaction was not statistically significant (*p* > 0.05). These data are shown in Table 4.

### 3.4. Oral Glucose Tolerance Test

**Figure 2:**
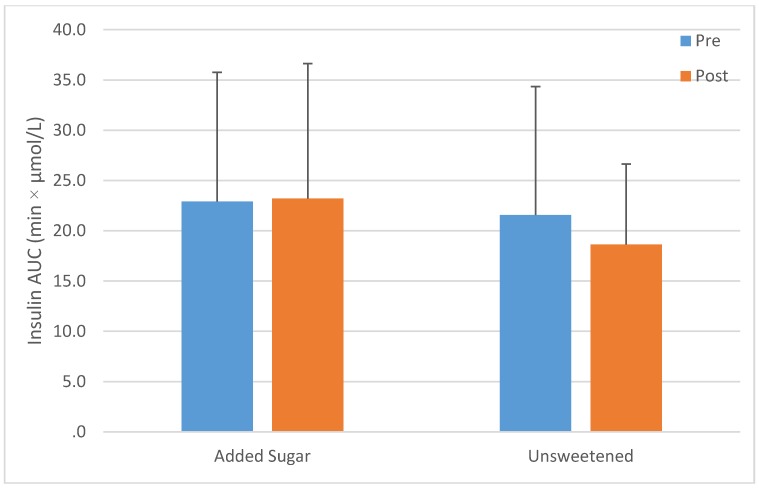
Change in insulin Area Under the Curve (AUC) after ten weeks’ consumption of low-fat milk sweetened with a sugar (*n* = 72) or unsweetened (*n* = 21); interaction *p* > 0.05.

**Figure 3:**
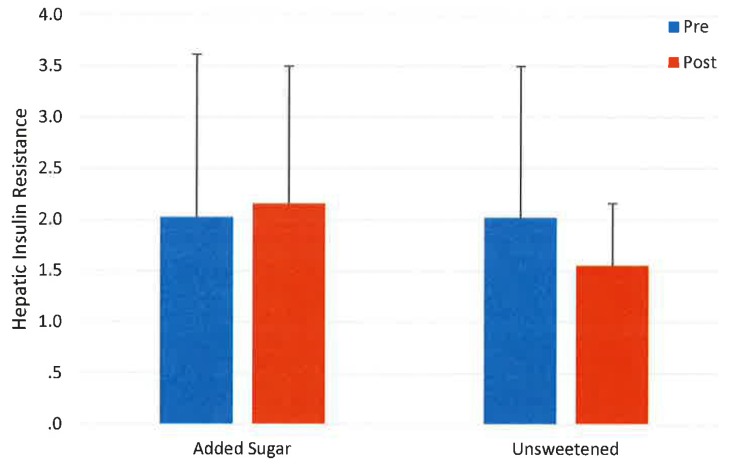
Change in hepatic insulin resistance after ten weeks’ consumption of low-fat milk sweetened with a sugar (*n* = 72) or unsweetened (*n* = 21); interaction *p* > 0.05.

There were no changes in glucose (760.4 ± 166.5 *vs.* 770.0 ± 165.5 min·mmol/L) or insulin AUC (22.9 ± 12.5 *vs.* 22.2 ± 12.5 min·µmol/L), in hepatic insulin resistance (2.0 ± 1.6 *vs.* 2.0 ± 1.2) or in ISI 16.0 ± 24.3 *vs.* 14.5 ± 26.6) in the entire pooled study population (*p* > 0.05). A time by group interaction was observed for both insulin AUC (*p* < 0.01) and hepatic insulin resistance (*p* < 0.05) In both cases there were no differences between the control milk and the entire pooled population of sweetened milk (Figure 2 and Figure 3). However, increases were observed in the fructose group (*p* < 0.012 and 0.05 respectively), but no other group. Both AUC and ISI were unchanged in the entire study population (*p* < 0.05) and no differences in responses were observed among the groups (interaction *p* > 0.05). These data are shown in Table 5.

**Table 4:** Fasting Measures obtained before and after ten weeks of daily consumption of sugar sweetened milk or unsweetened control milk.

		All	Time	HFCS 18%	Fructose 9%	Glucose 9%	Sucrose 18%	Control	Interaction
Body Weight (kg)	Pre	73.6 ± 13.0	<0.001	73.8 ± 13.2	74.1 ± 13.3	76.2 ± 12.2	72.1 ± 14.1	72.3 ± 12.2	0.191
	Post	74.5 ± 13.3		74.5 ± 13.8	74.7 ± 14.0	76.6 ± 12.1	73.4 ± 14.7	73.0 ± 12.4	
Glucose (mmol/L)	Pre	4.9 ± 0.4	0.056	5.1 ± 0.4	4.8 ± 0.4	5.0 ± 0.4	4.8 ± 0.4	5.0 ± 0.4	0.738
	Post	5.0 ± 0.5		5.2 ± 0.3	4.9 ± 0.7	5.0 ± 0.7	5.0 ± 0.4	5.0 ± 0.4	
Insulin (pmol/L)	Pre	56.9 ± 38.9	0.169	68.1 ± 36.8	55.6 ± 32.6	55.6 ± 32.6	54.2 ± 33.3	54.2 ± 35.4	0.133
	Post	61.8 ± 50.0		72.9 ± 51.4	79.2 ± 73.6	56.9 ± 52.1	55.6 ± 31.9	47.9 ± 28.5	
HOMA IR	Pre	1.8 ± 1.3	0.101	2.2 ± 1.2	1.7 ± 1.0	1.8 ± 1.7	1.7 ± 1.1	1.7 ± 1.2	0.112
	Post	2.0 ± 1.5		2.4 ± 1.8	2.4 ± 2.0	1.8 ± 1.3	1.8 ± 1.2	1.6 ± 1.0	

HFCS, high fructose corn syrup; HOMA IR, Homeostasis Model Assessment of Insulin Resistance.

**Table 5:** Oral Glucose Tolerance Measures obtained before and after ten weeks of daily consumption of sugar sweetened milk or unsweetened control milk.

		All	Time	HFCS 18%	Fructose 9%	Glucose 9%	Sucrose 18%	Control	Interaction
Glucose AUC (min·mmol/L)	Pre	760.4 ± 166.5	0.271	793.7 ± 194.3	760.4 ± 172.1	799.2 ± 155.4	749.3 ± 183.2	710.4 ± 138.8	0.969
	Post	777.0 ± 165.5		810.3 ± 194.3	782.6 ± 172.1	793.7 ± 144.3	765.9 ± 199.8	738.2 ± 127.7	
Insulin AUC (min·µmol/L)	Pre	22.9 ± 12.5	0.695	26.4 ± 18.8	19.4 ± 10.4	19.4 ± 8.3	26.4 ± 12.5	21.5 ± 12.5	0.005
	Post	22.2 ± 12.5		22.9 ± 13.9	25.7 ± 11.8 **	20.8 ± 16.0	23.6 ± 11.8	18.8 ± 8.3	
Matsuda Insulin	Pre	16.0 ± 24.3	0.681	21.3 ± 32.8	22.1 ± 45.8	12.9 ± 6.1	11.3 ± 6.8	14.7 ± 10.7	0.532
Sensitivity Index	Post	14.5 ± 26.6		28.0 ± 64.1	9.0 ± 4.2	12.4 ± 7.4	12.2 ± 10.0	13.8 ± 6.2	
Hepatic Insulin	Pre	2.0 ± 1.6	0.941	2.7 ± 2.6	1.5 ± 0.5	1.6 ± 0.7	2.4 ± 1.6	2.0 ± 1.5	0.032
Resistance	Post	2.0 ± 1.2		2.2 ± 1.3	2.1 ± 0.8 *	2.0 ± 1.5	2.4 ± 1.6	1.6 ± 0.6	

Different within group, *p* < 0.01 **, *p* < 0.05 *; HFCS = high fructose corn syrup; AUC, Area Under the Curve.

## 4. Discussion

These findings suggest that typically consumed fructose-containing sugars (e.g., sucrose and HFCS) when consumed at the median American intake level for added fructose do not produce changes in measures of insulin sensitivity or glucose tolerance when compared to a glucose control and an unsweetened beverage control. Furthermore, the identity of the added sugar, whether fructose itself, HFCS, or sucrose, or glucose, with minor exception is of limited importance. These findings are particularly important for the commonly-consumed sugars, sucrose and HFCS, which contain both fructose and glucose and do not appear to have deleterious effects on the parameters measured when consumed at average levels of intake.

These findings are consistent with a systematic review and meta-analysis of 18 trials of isocaloric exchange of fructose from other sources of carbohydrate by Cozma *et al.* [25]. These investigators reported that fructose consumption did not significantly increase fasting glucose or insulin and led to a reduction in hemoglobin A1C. Our findings are also consistent with those of Johnston *et al.* [41] who found in an acute experiment that fructose consumption, even at much higher levels than employed in the current trial, did not increase insulin resistance and was not different in this parameter from glucose.

Our findings are in contrast with those reported by Le *et al.* who found that four weeks of a high fructose diet did not affect insulin sensitivity or ectopic lipids in healthy humans [42]. Our findings are also consistent with the results from the InterAct European Consortium which showed that various digestible carbohydrates including sucrose were not associated with increased risk of type 2 diabetes in a large, prospective, cohort study involving 12,403 individuals with incident type 2 diabetes compared to a random sub-cohort of 16,835 individuals [14].

The increases in insulin AUC and hepatic insulin resistance found in the fructose group were unexpected. Since these changes were not found in either of the other groups who consumed fructose at similar levels together with glucose (*i.e.*, sucrose or HFCS), it is possible that the presence of glucose attenuated these responses. In particular, the co-ingestion of fructose with glucose facilitates the absorption of fructose although the mechanisms for this observation are not completely understood It is also possible that repeated exposure to pure fructose, an uncommon source of sugar in the human diet, may have led to these responses [43,44].

Our findings are in contrast with those reported by Silbernagel *et al.* who conducted a four week trial of very high fructose diet (150 grams of fructose a day), insulin sensitivity decreased [45]. Our findings are also different from a second trial by Le *et al.* who gave 3.5 grams per kilogram fat free mass of fructose to eight subjects who were offspring of individuals with type 2 diabetes and found decreased insulin sensitivity [7]. Results are also at variance with those by Stanhope *et al.* who gave 25% of calories from fructose compared to 25% of glucose and found increases in hepatic insulin resistance and total body insulin resistance [22]. It should be pointed out, however, that the experimental conditions in all of these latter studies were very different than the normal of levels and ways in which individuals typically consume fructose. For example, in the Stanhope trial, large amounts of pure fructose were compared to large amounts of glucose despite the fact that neither of these monosaccharides is consumed to any appreciable degree in the normal human diet. In contrast, our study employed the added sugars commonly consumed in the human diet (sucrose and HFCS) and found no differences or adverse effects from these sugars in the parameters measured. Our findings suggest that findings from the above referenced studies that compare the infrequently consumed monosaccharides, fructose and glucose, particularly at dosages above those normally consumed by humans, should be treated with great caution.

The mean fructose consumption in the American diet is 9% of calories. Thus, our goal was to deliver 9% of fructose. Since fructose by itself is rarely consumed in the diet, we also wanted to test the effects of 9% of fructose when consumed in typical sources, HFCS, and sucrose. Consumed in this form, 9% of calories as fructose requires 18% of calories from sucrose, and approximately the same amount from HFCS. We recognize that this involved giving a higher dose of sugar to these two groups. It is interesting to note, however, that despite receiving these higher doses of sugar in these groups the change in weight was comparable between the 9% and 18% sugar groups, and no differences among the groups were observed in fasting glucose, insulin, or insulin resistance (HOMA). This suggests that individuals are able to compensate for the additional calories from sugar at even at the 18% level by reducing caloric intake from other sources.

A number of epidemiologic studies have reported increased risk of type 2 diabetes associated with intake of sugar sweetened beverages (SSBs). Montonen *et al.* studied a cohort of 4304 men and women aged 40–60 years old who were free of diabetes at baseline [6]. These investigators demonstrated that HFCS and sucrose-sweetened beverages increase the risk of type 2 diabetes risk. De Koning *et al.* reported that participants in the top quartile of SSB intake had a 20% higher relative risk of diabetes than individuals in the bottom quartile [5]. Schulze *et al.* performed a prospective cohort analysis on the Nurses’ Health Study II and demonstrated that higher consumption of sugar sweetened beverages was associated with weight gain and increased risk of development of type 2 diabetes in women [8]. These investigators speculated that the increased risk of diabetes may have come from providing excess calories in large amounts of rapidly absorbable sugars. It should be emphasized that epidemiologic studies do not establish cause and effect and are considered lower quality evidence than randomized controlled trials such as the one reported here.

It should be pointed out that epidemiologic studies have also found an association between potato products [46] and red meat [47] and increased risk of type 2 diabetes. Thus, it remains in dispute whether or not sugar-sweetened beverages, *per se*, are associated with increased risk for diabetes or whether it is an overall nutritional pattern and level of caloric intake which may be associated with type 2 diabetes.

Several recent ecological studies have linked a rise in fructose availability with an increased risk of diabetes [9,10]. It should be pointed out, however, that ecological studies are considered low level evidence due to their limited ability to account for residual confounding. Furthermore, several other ecological studies, both in the United States and Australia [23,48], have not shown a positive trend between sugar intake and diabetes rate. In Australia, for example, despite a 10% decrease in sugar from SSBs, the prevalence of obesity and diabetes increased in line with other Western populations. This has been called the “Australian Paradox” [48]. In the United States, the prevalence of both obesity and diabetes have continued to rise over the past two decades despite decreases in consumption of added sugars [49,50]. Ecological studies, in particular, suffer from residual confounding due to heterogeneous measurement of exposure such as the use of population wide availability of HFCS or sucrose (*vs.* actual consumption) and imprecise databases and attempt to correlate this information to disease incidence. It is noteworthy that all five groups increased their energy intake comparably over the course of the 10 week intervention (*p* for time = 0.01; *p* for interaction 0.136). The changes in the sugar containing intervention groups were largely driven by carbohydrates, total sugar and added sugar; whereas, the increases in caloric consumption in the control condition were driven by increases in fat, carbohydrate, protein and total sugar. This suggests that individuals who increased the consumption of added sugar have no more difficulty incorporating that into their diet than individuals who increased caloric consumption from other sources.

Strengths of the current study include that it was a partially blinded, randomized, prospective controlled trial with a relatively large sample size which employed both glucose and a non-sweetened beverage controls. Furthermore, it utilized sugars that are typically consumed in the human diet. Weaknesses include that children and adolescents were excluded, as were subjects over the age of 60. Adolescents represent the single highest fructose consuming group in the United States [51]. Moreover, the subjects were only followed for ten weeks which should also be taken into consideration.

It should also be noted that the sugars were administered in milk. A meta-analysis of cohort studies has recently suggested that dairy products may decrease the risk of type 2 diabetes although the mechanism of this decrease is not fully understood [52]. It has been postulated that milk proteins or other components of milk may contribute to decreased risk of diabetes. It should be noted, however, that all five groups including the four groups that consumed added sugar in low-fat milk and the control group which contained unsweetened low-fat milk consumed similar amounts of milk which makes it unlikely that the consumption of milk altered our results in any appreciable way. Nonetheless, the possibility that utilization of milk as a delivery vehicle may have attenuated the glucose and insulin responses in all groups. This should be taken into consideration when interpreting our results.

A further limitation of the study relates to the unstructured nature of the diet. This means that the study was only able to control the amount of added sugar (HFCS, fructose, glucose or sucrose) consumed in the milk provided. This means that all participants consumed additional sugars in amounts and of an identity that were not controlled. Sufficient information is not available on the amount of different sugars contained in the majority of commercially produced foods; therefore, we were unable to measure the amount of HFCS, fructose, glucose, and sucrose actually consumed. However, we made the assumption that the additional sugar consumed by each participant had was comparable among the groups in terms of amounts and identity (predominantly HFCS and sucrose). As seen in Table 4, the result was that the two 18% groups consumed a higher percentage of calories from sugar and added sugar than the 9% groups and the unsweetened control, and also the interventional sugars provided the majority of added sugar consumed for all groups.

## 5. Conclusions

In conclusion, findings from this randomized controlled trial suggest that fructose containing sugars consumed at the 50th percentile population consumption level do not increase insulin resistance or decrease glucose tolerance. When given at median levels, consumption levels fell over a ten week period. This study contributes to the growing literature that at typical human consumption levels, intake of the most commonly consumed sugars do not appear to increase risk factors for diabetes at least over a 10-week trial period.

## References

[1] Hu F.B. (2011). Globalization of diabetes: The role of diet, lifestyle, and genes. Diabetes Care.

[2] Shaw J.E., Sicree R.A., Zimmet P.Z. (2010). Global estimates of the prevalence of diabetes for 2010 and 2030. Diabetes Res. Clin. Pract..

[3] Danaei G., Finucane M.M., Lu Y., Singh G.M., Cowan M.J., Paciorek C.J., Lin J.K., Farzadfar F., Khang Y.H., Stevens G.A. (2011). National, regional, and global trends in fasting plasma glucose and diabetes prevalence since 1980: Systematic analysis of health examination surveys and epidemiological studies with 370 country-years and 2.7 million participants. Lancet.

[4] International Diabetes Federation Epidemiology and Morbidity. IDF Diabetes Atlas.

[5] De Koning L., Malik V.S., Kellogg M.D., Rimm E.B., Willett W.C., Hu F.B. (2012). Sweetened beverage consumption, incident coronary heart disease and biomarkers of risk in men. Circulation.

[6] Montonen J., Jarvinen R., Knekt P., Heliovaara M., Reunanen A. (2007). Consumption of sweetened beverages and intakes of fructose and glucose predict type 2 diabetes occurrence. J. Nutr..

[7] Le K.A., Ith M., Kreis R., Faeh D., Bortolotti M., Tran C., Boesch C., Tappy L. (2009). Fructose overconsumption causes dyslipidemia and ectopic lipid deposition in healthy subjects with and without a family history of type 2 diabetes. Am. J. Clin. Nutr..

[8] Schulze M.B., Hoffmann K., Manson J.E., Willett W.C., Meigs J.B., Weikert C., Heidemann C., Colditz G.A., Hu F.B. (2005). Dietary pattern, inflammation, and incidence of type 2 diabetes in women. Am. J. Clin. Nutr..

[9] Goran M.I., Ulijaszek S.J., Ventura E.E. (2013). High fructose corn syrup and diabetes prevalence: A global perspective. Global Public Health.

[10] Basu S., Yoffe P., Hills N., Lustig R.H. (2013). The relationship of sugar to population-level diabetes prevalence: An econometric analysis of repeated cross-sectional data. PLoS ONE.

[11] Bray G.A. (2012). Fructose and risk of cardiometabolic disease. Curr. Atheroscler. Rep..

[12] Johnson R., Segal M., Sautin Y., Nakagawa T., Feig D.I., Kang D.H., Gersch M.S., Benner S., Sanchez-Lozada L.G. (2007). Potential role of sugar (fructose) in the epidemic of hypertension, obesity and the metabolic syndrome, diabetes, kidney disease, and cardiovascular disease. Am. J. Clin. Nutr..

[13] Sanchez-Lozada L.G., Le M., Segal M., Johnson R.J. (2008). How safe is fructose for persons with or without diabetes?. Am. J. Clin. Nutr..

[14] The InterAct Consortium (2013). Consumption of sweet beverages and type 2 diabetes incidence in European adults: Results from EPIC-InterAct. Diabetologia.

[15] Sluijs I., Beulens J.W.J., van der Schouw Y.T., van der A D.L., Buckland G., Kuijsten A., Schulze M.B., Amiano P., Ardanaz E., Balkau B. (2013). Dietary glycemic index, glycemic load, and digestible carbohydrate intake are not associated with risk of type 2 diabetes in eight European countries. J. Nutr..

[16] Pooranaperundevi M., Sumiyabanu M.S., Viswanathan P., Sundarapandiyan R., Anuradha C.V. (2010). Insulin resistance induced by high-fructose diet potentiates carbon tetrachloride hepatotoxicity. Toxicol. Ind. Health.

[17] Lewis G.F., Uffelman K., Naples M., Szeto L., Haidari M., Adeli K. (2005). Intestinal lipoprotein overproduction, a nearly recognized component of insulin resistance, is ameliorated by the insulin sensitizer rosiglitazone: Studies in the fructose-fed Syrian golden hamster. Endocrinology.

[18] Thorburn A.W., Storlien L.H., Jenkins A.B., Khouri S., Kraegen E.W. (1989). Fructose-induced *in vivo* insulin resistance and elevated plasma triglyceride levels in rats. Am. J. Clin. Nutr..

[19] Rizkalla S.W., Boillot J., Tricottet V., Fontvieille A.M., Luo J., Salzman J.L., Camilleri J.P., Slama G. (1993). Effects of chronic dietary fructose with and without copper supplementation on glycaemic control, adiposity, insulin binding to adipocytes and glomerular basement membrane thickness in normal rats. Br. J. Nutr..

[20] Maiztegui B., Borelli M.I., Raschia M.A., Del Zotto H., Gagliardino J.J. (2009). Islet adaptive changes to fructose-induced insulin resistance: Beta-cell mass, glucokinase, glucose metabolism, and insulin secretion. J. Endocrinol..

[21] Mielke J.G., Taghibiglou C., Liu L., Zhang Y., Jia Z., Adeli K., Wang Y.T. (2005). A biochemical and functional characterization of diet-induced brain insulin resistance. J. Neurochem..

[22] Stanhope K.L., Schwarz J.M., Keim N.L., Griffen S.C., Bremer A.A., Graham J.L., Hatcher B., Cox C.L., Dyachenko A., Zhang W. (2009). Consuming fructose-sweetened, not glucose-sweetened, beverages increases visceral adiposity and lipids and decreases insulin sensitivity in overweight/obese humans. J. Clin. Investig..

[23] Cozma A.I., Ha V., de Souza R.J., Sievenpiper J., Rippe J.M. (2014). Sweeteners and Diabetes. Fructose, High Fructose Corn Syrup, Sucrose and Health.

[24] Saris W.H., Astrup A., Prentice A.M., Zunft H.J., Formiguera X., Verboeket-van de Venne W.P., Raben A., Poppitt S.D., Seppelt B., Johnston S. (2000). Randomized controlled trial of changes in dietary carbohydrate/fat ratio and simple *vs.* complex carbohydrates on body weight and blood lipids: The CARMEN study. The Carbohydrate Ratio Management in European National diets. Int. J. Obes. Relat. Metab. Disord..

[25] Cozma A.I., Sievenpiper J.L., de Souza R.J., Chiavaroli L., Ha V., Wang D.D., Mirrahimi A., Yu M.E., Carleton A.J., Di Buono M. (2012). Effect of fructose on glycemic control in diabetes: A systematic review and meta-analysis of controlled feeding trials. Diabetes Care.

[26] Colditz G.A., Manson J.E., Stampfer M.J., Rosner B., Willett W.C., Speizer F.E. (1992). Diet and risk of clinical diabetes in women. Am. J. Clin. Nutr..

[27] Meyer K.A., Kushi L.H., Jacobs D.R., Slavin J., Sellers T.A., Folsom A.R. (2000). Carbohydrates, dietary fiber, and incident type 2 diabetes in older women. Am. J. Clin. Nutr..

[28] Janket S.J., Manson J.E., Sesso H., Buring J.E., Liu S. (2003). A prospective study of sugar intake and risk of type 2 diabetes in women. Diabetes Care.

[29] Hodge A.M., English D.R., O’Dea K., Giles G.G. (2004). Glycemic index and dietary fiber and the risk of type 2 diabetes. Diabetes Care.

[30] Morino K., Petersen K.F., Shulman G.I. (2006). Molecular mechanisms of insulin resistance in humans and their potential links with mitochondrial dysfunction. Diabetes.

[31] Seppala-Lindroos A., Vehkavaara S., Hakkinen A.M., Goto T., Westerbacka J., Sovijarvi A., Halavaara J., Yki-Jarvinen H. (2002). Fat accumulation in the liver is associated with defects in insulin suppression of glucose production and serum free fatty acids independent of obesity in normal men. J. Clin. Endocrinol. Metab..

[32] Hellerstein M.K., Schwarz J.M., Neese R.A. (1996). Regulation of hepatic *de novo* lipogenesis in humans. Annu. Rev. Nutr..

[33] Hellerstein M.K. (2001). No common energy currency: *De novo* lipogenesis as the road less traveled. Am. J. Clin. Nutr..

[34] Peterson K.F., Shulman G.I. (2007). Etiology of insulin resistance. Am. J. Med..

[35] Shulman G.I. (2014). Ectopic fat in insulin resistance, dyslipidemia, and cardiometabolic disease. N. Engl. J. Med..

[36] Lowndes J., Kawiecki D., Yu Z., Rippe J.M. (2015). No Dose Response Relationship in the Effects of Commonly Consumed Sugars on Risk Factors for Diabetes across a Range of Typical Human Consumption Levels. Food Nutr. Sci..

[37] Mifflin M.D., St. Jeor S.T., Hill L.A., Scott B.J., Daugherty S.A., Koh Y.O. (1990). A new predictive equation for resting energy expenditure in healthy individuals. Am. J. Clin. Nutr..

[38] Matthews D.R., Hosker J.P., Rudenski A.S., Naylor B.A., Treacher D.F., Turner R.C. (1985). Homeostasis model assessment: Insulin resistance and beta-cell function from fasting plasma glucose and insulin concentrations in man. Diabetologia.

[39] Abdul-Ghani M.A., Matsuda M., Balas B., DeFronzo R.A. (2007). Muscle and liver insulin resistance indexes derived from the oral glucose tolerance test. Diabetes Care.

[40] Matsuda M., DeFronzo R.A. (1999). Insulin sensitivity indices obtained from oral glucose tolerance testing: Comparison with the euglycemic insulin clamp. Diabetes Care.

[41] Johnston R.D., Stephenson M.C., Crossland H., Cordon S.M., Palcidi E., Cox E.F., Taylor M.A., Aithal G.P., MacDonald I.A. (2013). No difference between high fructose and high glucose diets on liver tricylglycerol or biochemistry in healthy overweight men. Gastroenterology.

[42] Lê K.-A., Faeh D., Stettler R., Ith M., Kreis R., Vermathen P., Boesch C., Ravussin E., Tappy L. (2006). A 4-wk high-fructose diet alters lipid metabolism without affecting insulin sensitivity or ectopic lipids in healthy humans. Am. J. Clin. Nutr..

[43] Latulippe M.E., Skoog S.M. (2011). Fructose malabsorption and intolerance: Effects of fructose with and without simultaneous glucose ingestion. Crit. Rev. Food Sci. Nutr..

[44] Tappy L., Egli L., Tran C., Rippe J.M. (2014). Metabolism of Nutritive Sweeteners in Humans. Fructose, High Fructose Corn Syrup, Sucrose and Health.

[45] Silbernagel G., Machann J., Unmuth S., Schick F., Stefan N., Hans U., Ring H., Fritsche A. (2011). Effects of 4-week very-high-fructose/glucose diets on insulin sensitivity, visceral fat and intrahepatic lipids: An exploratory trial. Br. J. Nutr..

[46] Pan A., Sun Q., Bernstein A.M., Schulze M.B., Manson J.E., Willett W.C., Hu F.B. (2011). Red meat consumption and risk of type 2 diabetes: 3 Cohorts of US adults and an updated meta-analysis. Am. J. Clin. Nutr..

[47] Halton T.L., Willett W.C., Liu S., Manson J.E., Stampfer M.J., Hu F.B. (2006). Potato and French fry consumption and risk of type 2 diabetes in women. Am. J. Clin. Nutr..

[48] Barclay A.W., Brand-Miller J. (2012). The Australian paradox: A substantial decline in sugars intake over the same timeframe that overweight and obesity have increased. Nutrients.

[49] Centers for Disease Control and Prevention Total added sugars, have not resulted in a decrease of obesity or diabetes in the US. http://www.cdc.gov/diabetes/statistics/slides/maps_diabetesobesity_trends.pdf.

[50] Welsh J.A., Sharma A.J., Grellinger L., Vos M.B. (2011). Consumption of added sugars is decreasing in the United States. Am. J. Clin. Nutr..

[51] Marriott B., Cole N., Lee E. (2009). National estimates of dietary fructose intake increased from 1977 to 2004 in the United States. J. Nutr..

[52] Dabfinn A., Norat T., Romundstad P., Vatten L.J. (2013). Dairy products and the risk of type 2 diabetes: A systematic review and dose-response meta-analysis of cohort studies. Am. J. Clin. Nutr..

